# Fibroblast Growth Factor Signaling Potentiates VE-Cadherin Stability at Adherens Junctions by Regulating SHP2

**DOI:** 10.1371/journal.pone.0037600

**Published:** 2012-05-22

**Authors:** Kunihiko Hatanaka, Anthony A. Lanahan, Masahiro Murakami, Michael Simons

**Affiliations:** 1 Section of Cardiovascular Medicine, Department of Internal Medicine, Yale University School of Medicine, New Haven, Connecticut, United States of America; 2 Department of Cell Biology, Yale University School of Medicine, New Haven, Connecticut, United States of America; University of Illinois at Chicago, United States of America

## Abstract

**Background:**

The fibroblast growth factor (FGF) system plays a critical role in the maintenance of vascular integrity via enhancing the stability of VE-cadherin at adherens junctions. However, the precise molecular mechanism is not well understood. In the present study, we aimed to investigate the detailed mechanism of FGF regulation of VE-cadherin function that leads to endothelial junction stabilization.

**Methods and Findings:**

In vitro studies demonstrated that the loss of FGF signaling disrupts the VE-cadherin-catenin complex at adherens junctions by increasing tyrosine phosphorylation levels of VE-cadherin. Among protein tyrosine phosphatases (PTPs) known to be involved in the maintenance of the VE-cadherin complex, suppression of FGF signaling reduces SHP2 expression levels and SHP2/VE-cadherin interaction due to accelerated SHP2 protein degradation. Increased endothelial permeability caused by FGF signaling inhibition was rescued by SHP2 overexpression, indicating the critical role of SHP2 in the maintenance of endothelial junction integrity.

**Conclusions:**

These results identify FGF-dependent maintenance of SHP2 as an important new mechanism controlling the extent of VE-cadherin tyrosine phosphorylation, thereby regulating its presence in adherens junctions and endothelial permeability.

## Introduction

Regulation of endothelial permeability is crucial for many essential vascular functions including the passage of molecules and cells through the endothelium without altering structural integrity of blood vessels [1], [2].

The maintenance of the vascular barrier function is largely achieved by endothelial cell junctions which are comprised of a complex network of adhesive proteins organized into tight junctions and adherens junctions [3], [4]. The formation of adherens junctions is required for the correct organization of tight junctions; therefore, assembly and disassembly of adherens junction is strictly controlled and critically important for the overall endothelial homeostasis [5]. Among molecules localized at adherens junctions, VE-cadherin, a transmembrane homophilic adhesion receptor, plays a key role in this regulation. Although VE-cadherin has been regarded as primarily involved in mediating intercellular adhesion and controlling vascular permeability, recent studies began to reveal its more diverse involvement in a wide variety of vascular functions [6], [7]. VE-cadherin interacts, via its cytoplasmic domain, with three proteins of the armadillo family: p120-catenin, β-catenin, and plakoglobin. p120-catenin binds VE-cadherin at the juxtamembrane domain of its cytoplasmic tail, preventing internalization and degradation of VE-cadherin, thereby maintaining cell-cell adhesion [8]. Permeability-increasing agents such as histamine, tumor necrosis factor-alpha, platelet-activating factor, and vascular endothelial growth factor (VEGF) induce phosphorylation of the VE-cadherin-catenin complex [9], [10], [11], [12], [13]. Src-induced phosphorylation of Y658 or Y731 of VE-cadherin prevents binding of p120-catenin and β-catenin, respectively, which increases endothelial permeability and is sufficient to maintain cells in a mesenchymal state [14], [15]. Moreover, phosphorylation of specific VE-cadherin tyrosines is also induced by leukocyte adhesion to the endothelium via intercellular adhesion molecule 1 (ICAM-1), facilitating leukocyte transmigration [16], [17]. Phosphorylation of VE-cadherin appears to be tightly controlled by both kinases and phosphatases. Several protein tyrosine phosphatases (PTPs) including DEP-1, VE-PTP (PTPβ), PTPµ, PTP1B and SHP2 are capable of dephosphorylating VE-cadherin or associating proteins and are implicated in functional modification of the VE-cadherin-catenin complex [18], [19], [20], [21], [22].

We have recently found, using in vitro and in vivo approaches, that inhibition of fibroblast growth factor (FGF) signaling impairs vascular integrity in the adult vasculature [23]. Specifically, the lack of endothelial FGF signaling leads to dissociation of p120-catenin from VE-cadherin and displacement of VE-cadherin from cell-cell contacts. This, in turn, progresses to the disorganization of endothelial cell junctions, leading to severe impairment of endothelial barrier function. In this study, we investigated molecular mechanisms involved in FGF-dependent regulation of VE-cadherin phosphorylation and permeability control. We found that FGF signaling controls VE-cadherin phosphorylation by regulating SHP2 expression and function rather than modifying the activity of VE-cadherin kinases. The absence of FGF signaling leads to impaired SHP2 expression and reduces its binding to VE-cadherin which, in turn, enhances tyrosine phosphorylation of VE-cadherin including the Y658 site required for VE-cadherin-p120-catenin interaction. This defect was fully reversed by SHP2 overexpression in endothelial cells with suppressed FGF signaling. We conclude, therefore, that FGF signaling potentiates VE-cadherin stability at adherens junctions by regulating SHP2 expression.

## Methods

### Reagents and Antibodies

Antibodies against the following antigens were commercially obtained: VE-cadherin, p120-catenin, SHP2, PTPβ, DEP-1, PTP1B, c-Src (Santa Cruz Biotechnology), PTPµ, phospho-tyrosine 416 Src, phospho-tyrosine 527 Src, Fak, phospho tyrosine 397 Fak (Cell Signaling technology), phospho-tyrosine 658 VE-cadherin, phospho-tyrosine 731 VE-cadherin (invitrogen), HA (COVANCE), and β-tubulin (Sigma). Anti phospho-tyrosine 685 VE-cadherin antibody was generated in our laboratory.

### Cell Culture and Adenoviral Transduction

Bovine aortic endothelial cells (BAEC) and Human umbilical vein endothelial cells (HUVEC) were purchased from Lonza, and were cultured at 37°C in 5% CO_2_ in EGM-2 medium (Lonza). Adenovirus carrying the truncated form of FGF receptor 1 lacking the cytoplasmic part of the receptor (FGFR1DN) was generated as previously described [23]. Adenovirus carrying PTPN11 (SHP2) was obtained from VECTOR BIOLABS. Adenoviral vectors were transduced at a multiplicity of infection (MOI) of 10–100 vp/cell. The infection medium was replaced 6 hours later with normal growth medium. For soluble FGFR (sFGFR) experiments, adenovirus encoding GFP (control), sFGFR1-IIIc or sFGFR3-IIIb was transduced in BAEC [23], conditioned media from transduced BAEC were collected two days later, then transferred to uninfected BAEC. Total cell lysates were extracted 24 hrs after medium transfer.

### GFP-tagged VE-cadherin Construct and Transfection of Endothelial Cells

cDNA of human VE-cadherin fused in-frame with GFP at the C-terminus (VE-cadherin-GFP) was a kind gift from Dr. Sunil K. Shaw (Women and Infants’ Hospital, Providence, RI) [24]. VE-cadherin-GFP was subcloned into pcDNA3.1 (Invitrogen). A tyrosine to phenylalanine (Y to F) mutation at VE-cadherin Y658 site (Y658F) was introduced using the QuickChange site-directed mutagenesis kit (Stratagene). Plasmids were transfected into BAEC using FuGENE 6 Transfection Reagent (Roche) following the manufacture’s instruction.

### Lentivirus Vector Construct and Transduction of Endothelial Cells

Human SHP2 constructs (wild-type and C/S mutant) were purchased from Addgene and subcloned into pLVX-IRES-puro lentiviral expression vector (Clontech). Lentivirus packaging and envelope vectors, pMDLg/pRRE, pRSV-Rev and pMD2.G, were also purchased from Addgene. These plasmids were cotransfected into HEK-293T cells to produce a recombinant lentivirus stock [25].

BAEC on a 6-well plate were cultured in EGM-2 medium (Lonza) to 50–60% confluent. Lentivirus was added to the cells with 5 µg/ml polybrene and the 6-well plate was centrifuged at 2300 rpm for 90 min at 37°C following an 8-hour incubation at 37°C with 5% CO_2_. To obtain stable infected cells, the cells were selected with 1 µg/ml puromycin.

### Immunofluorescence

Immunocytochemistry was performed with a standard procedure using antibodies described above. Cells were fixed in 2% paraformaldehyde in phosphate buffered saline (PBS) for 15 min at room temperature (RT), permeabilized in 0.1% Triton-X in PBS for 5 min at RT, and blocked in Image-iT FX signal enhancer (Invitrogen) for 30 min at RT. Coverslips were incubated in primary antibody at 4°C overnight, washed three times in PBS and then incubated with Alexa Fluor-conjugated secondary antibodies (Invitrogen) for 1 hour at RT. Then the coverslips were washed three times in PBS and mounted using Fluoro-Gel (Electron Microscopy Sciences). Images were captured using Zeiss 510 laser scanning confocal microscopy. Gap formation of the monolayer was analyzed using immunostaining images in which the proportion of the gap area between cells to the total image area was calculated using NIH Image J software.

### Permeability Assay

Transendothelial electrical resistance, an index of endothelial cell barrier function, was measured in real time using an electric cell-substrate impedance sensing (ECIS) system (ECIS 1600, Applied BioPhysics) [15], [23]. Cells were plated on sterile 8-chambered gold-plated electrode arrays (8W10E plus) precoated with fibronectin and grown to full confluence. The electrode arrays were mounted on the ECIS system within an incubator (37°C, 5% CO_2_) and connected to its recorder device. Monolayer resistance was recorded at 15 kHz in 5-minute intervals.

### Western Blotting and Immunoprecipitation

Cells were washed twice with ice-cold PBS and lysed in RIPA buffer: 50 mM Tris-HCl, pH 7.4, 150 mM NaCl, 1% NP40, 0.5% sodium deoxycholate, 0.1% SDS, and 1× Complete protease inhibitor mixture (Roche) and PhosSTOP Phosphatase inhibitor cocktail (Roche). For evaluation of VE-cadherin phosphorylation, lysis buffer was supplemented with freshly prepared pervanadate (300 µM) [26]. Whole cell lysates were subjected to SDS-PAGE followed by immunoblotting with the antibodies described above. For immunoprecipitation assays, whole cell lysates were harvested with HEPES lysis buffer with NP-40 (50 mM HEPES pH 7.4, 150 mM NaCl, 1% NP-40, 5 mM EDTA and 1× Complete protease inhibitor mixture, (Roche) and PhosSTOP Phosphatase inhibitor cocktail (Roche) supplemented with 300 µM pervanadate and incubated with the appropriate antibody and Dynabeads Protein G (Invitrogen). Isotype-matched IgG was used as a negative control. Beads were washed 4 times with PBS, boiled in SDS-PAGE loading buffer, and resolved by SDS-PAGE [27].

### Quantitative Real-time PCR

RNA preparation and cDNA synthesis were described previously [28]. Total RNA was isolated with an RNeasy Plus Mini Kit (Qiagen), and cDNA was synthesized using a High Capacity cDNA Reverse Transcription Kit (Applied Biosystems). PCR amplification was carried out with gene specific primers: bovine SHP2, forward 5′-TGATTCGCTGTCAGGAACTG; reverse 5′-TGTGCCCAATGTTTCTACCA-3′, bovine GAPDH, forward 5′-GGCGCCAAGAGGGTCAT-3′; reverse 5′-GTGGTTCACGCCCATCACA-3′, using Ssofast EvaGreen Supermix (Bio-Rad) on a CFX96™ Real-Time PCR Detection System.

### Statistics

Data were expressed as mean ± SD. Comparison between groups were performed with a two-tailed Student’s *t* test. Results were considered significant at *P*<0.05.

## Results

### Disruption of FGF Signaling Affects Endothelial Monolayer Integrity and Permeability

**Figure 1:**
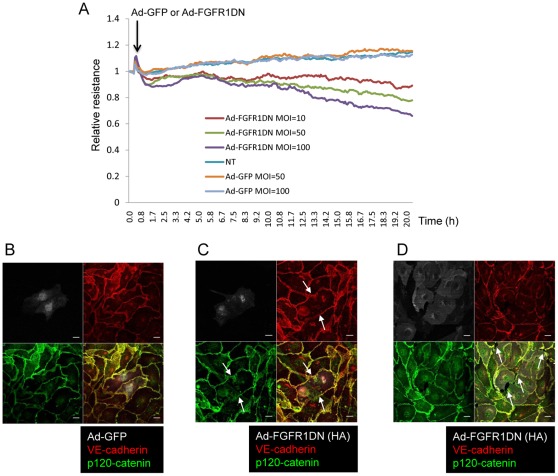
FGF signaling is necessary for endothelial cell monolayer integrity. **A.** Endothelial monolayer permeability evaluated with the ECIS system. Transendothelial resistance was measured every 5 minutes for 20 hours after the onset of adenoviral transduction. The experiment reported has been performed 3 times with comparable results. Note that cells lacking FGF signaling showed decreased transendothelial resistance (increased endothelial permeability). **B, C, D.** VE-cadherin was absent from cell-cell contacts in cells lacking FGF signaling. Immunostaining of quiescent and fully confluent BAEC transduced with Ad-GFP (B) or Ad-FGFR1DN (C: MOI = 10, D: MOI = 100). Cells were stained for VE-cadherin (red) and p120-catenin (green). Note that VE-cadherin was absent from cell-cell contacts (arrows in C) and many gaps were formed (arrows in D) in the monolayer transduced with Ad-FGFR1DN. Scale Bars: 10 µm.

To investigate the role of FGF signaling in the vasculature, we employed a dominant negative construct of FGF receptor 1 (FGFR1DN) capable of heterodimerizing with all four isoforms of FGF receptors, thereby inhibiting overall FGF signaling [29]. First, we tested the effect of suppression of FGF signaling on endothelial permeability. Ad-FGFR1DN transduction of BAEC resulted in a dose-dependent decrease in transendothelial resistance compared with control cells (Fig. 1A). Adherens junctions in the endothelium play a central role in regulation of vascular permeability. Since VE-cadherin is the principal protein regulating not only adherens junction formation, but also endothelial permeability by opening paracellular junctions, we evaluated VE-cadherin expression and distribution in cells lacking FGF signaling. While VE-cadherin and p120-catenin localize at cell-cell contacts in the control BAEC monolayer, (Fig. 1B), BAEC transduced with Ad-FGFR1DN showed the loss of VE-cadherin and p120-catenin from cell-cell junctions (Fig. 1C, arrows). Exposure of BAEC with a higher dose of Ad-FGFR1DN resulted in the gap formation between endothelial cells (Fig. 1D, arrows). No gap formation was observed in Ad-GFP transduced cells (Fig. 1B). Thus, basal FGF signaling is necessary for VE-cadherin stability at adherens junctions.

### FGF Signaling Regulates VE-cadherin-p120-catenin Association through Modulating VE-cadherin Phosphorylation Level

**Figure 2:**
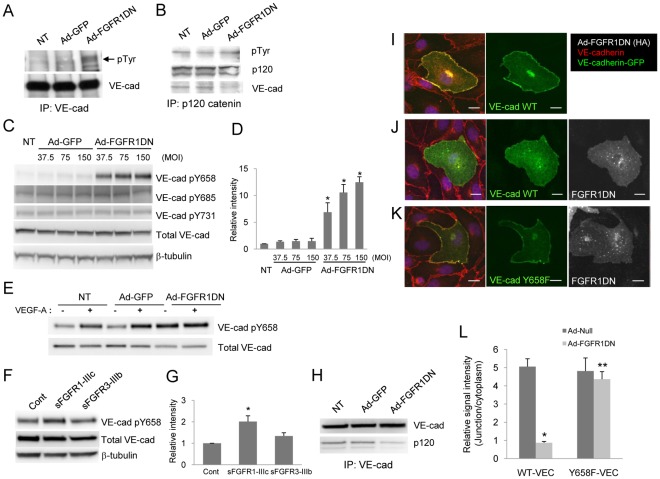
Suppression of FGF signaling increases Y658 phosphorylation of VE-cadherin and disrupts VE-cadherin/p120-catenin association. **A**. Increased VE-cadherin tyrosine phosphorylation in cells lacking FGF signaling. BAEC were transduced with Ad-GFP or Ad-FGFR1DN and cell lysates were immunoprecipitated (IP) with VE-cadherin antibody. Phosphorylated VE-cadherin was immunoblotted (IB) with phosphotyrosine-specific (pY20) antibody. The same membrane was reprobed for VE-cadherin. NT denotes non-transduction. **B**. Tyrosine phosphorylation of p120-catenin was not affected by FGF inhibition in endothelial cells. BAEC were transduced with Ad-GFP or Ad-FGFR1DN and cell lysates were immunoprecipitated (IP) with p120-catenin antibody. Phosphorylated p120-catenin was evaluated with phosphotyrosine-specific (pY20) antibody. The same membrane was reprobed for p120-catenin and VE-cadherin. **C**. Phosphorylation of VE-cadherin Y658 was increased in cells with depleted FGF signaling. BAEC were transduced with Ad-GFP or Ad-FGFR1DN. Western blot analysis of total cell lysate using phospho-specific antibodies shows Y658 phosphorylation was increased in Ad-FGFR1DN transduced cells. **D**. Quantitative analysis of pY658 VE-cadherin shown in Fig. 2C. The value of NT, standardized with β-tubulin, was designated as 1. (n = 3 Mean ± SD, *P<0.01, by t-test compared with NT control). **E**. Confluent BAEC untreated or transduced with either Ad-GFP or Ad-FGFR1DN were starved with 0.5% FBS in EBM-2 for 16 hours and then were stimulated with 50 ng/ml VEGF-A for 15 mins. Total cell lysates were subjected to Western analysis and probed for indicated antibodies. **F**. Confluent BAEC monolayers were treated with medium containing FGF-trap (sFGFR1-IIIc or sFGFR3-IIIb), and total cell lysates were subjected to Western blotting. Medium collected from Ad-GFP transduced cells was used as control. **G**. Quantitative analysis of pY658 VE-cadherin shown in Fig. 2F. The value of control, standardized with β-tubulin, was designated as 1. (n = 3, Mean ± SD, *P<0.05, by t-test compared with control). **H**. Decreased p120-catenin binding to VE-cadherin in cells lacking FGF signaling. BAEC were transduced with Ad-GFP or Ad-FGFR1DN. Cells were lysed and VE-cadherin was immunoprecipitated (IP). Immunoprecipitates were subjected to SDS-PAGE followed by immunoblotting (IB) with the indicated antibodies. NT denotes no transduction. **I**, **J**, **K**. Y658F mutation of VE-cadherin rescued the FGFR1DN phenotype. VE-cadherin-GFP constructs (wild-type or Y658F mutant) were transfected into HUVEC which were transduced with either Ad-Null or Ad-FGFR1DN. Cells were stained for VE-cadherin (red), VE-cadherin-GFP (green) and Ad-FGFR1DN (white). Note that wild-type VE-cadherin-GFP was absent from cell-cell contacts in cells lacking FGF signaling (J) while Y658F VE-cadherin localized at endothelial junctions in the absence of FGF signaling (K). Scale Bars: 10 µm. **L**. Quantitative analysis of VE-cadherin distribution. Distribution of transfected VE-cadherin-GFP was evaluated by measuring GFP signal intensity of a 3 µm^2^ area set either at cell-cell junction (J) or cytoplasm (C, adjacent to cell-cell junction, not including Golgi) using Volocity software (Perkin Elmer). In each cell, six-seven measuring areas were set at J and C region, respectively, and total six cells were evaluated in each treatment group. The data is shown as a ratio of J/C, mean ± SEM. *: *P*<0.01 vs. Wild type VE-cadherin/Ad-Null, **: *P*<0.01, vs. Wild type VE-cadherin/Ad-FGF-R1DN by t-test.

Previous reports support the idea that VE-cadherin stability at adherens junctions is dependent on the phosphorylation status of VE-cadherin, which modulates VE-cadherin/p120-catenin interaction required for the retention of the VE-cadherin complex at cell-cell contacts [8], [30]. As shown in Fig. 2A, shutdown of FGF signaling resulted in enhanced tyrosine phosphorylation of VE-cadherin. In contrast, p120 tyrosine phosphorylation was not affected by FGF inhibition, suggesting that FGF signaling primarily controls endothelial permeability through modulating VE-cadherin phosphorylation (Fig. 2B). In the cytoplasmic tail of VE-cadherin, at least five tyrosine (Y645, Y658, Y685, Y731 and Y733) and one serine (S665) residues can be phosphorylated *in vitro* and are thought to be involved in the regulation of endothelial permeability and leukocyte transmigration [14], [17], [31], [32], [33]. Dephosphorylation of Y658 is essential for p120-catenin binding and VE-cadherin stability at adherens junctions [14], [15]. Therefore, we wished to identify the tyrosine site involved in the FGF-mediated VE-cadherin stabilization. Western blotting with phospho-specific antibodies demonstrated that suppression of FGF signaling increased Y658 phosphorylation while phosphorylation of Y685 and Y731 sites was not affected (Fig. 2C, D). Since it has been demonstrated that VEGF-A is capable of phosphorylating Y658 in the cytoplasmic domain of VE-cadherin, thus disrupting the VE-cadherin complex in adherens junctions [14], we examined the effect of VEGF-A in endothelial cells defective in FGF signaling. Although VEGF-A induced Y658 VE-cadherin phosphorylation in either non-transduced or Ad-GFP transduced endothelial cells as has been reported previously [34], [35], Y658 phosphorylation in Ad-FGFR1DN transduced cells was increased at baseline and no further induction was observed after VEGF-A stimulation (Fig. 2E). While this observation is consistent with our result in Fig. 2A and 2C, cancellation of the VEGF effect in FGFR1DN cells may be due to VEGFR2 downregulation in endothelial cells lacking FGF signaling that has been demonstrated in the previous study [36].

To further confirm the FGF effect on VE-cadherin phosphorylation, we employed a soluble FGF receptor (FGF-trap) as an alternative means to inhibit FGF signaling. sFGFR has been extensively evaluated and shown to be an effective inhibitor of FGF signaling both in vitro and in vivo [23]. Using the conditioned medium containing sFGFR1-IIIc which has the capacity to bind multiple FGF ligands, we were able to reproduce the results obtained using Ad-FGFR1DN. In contrast, sFGFR3-IIIb which binds a very small subset of FGF ligands with low affinities and does not affect vascular permeability had little effect on Y658 VE-cadherin phosphorylation (Fig. 2F, G) [37]. In line with these observations, co-immunoprecipitation experiments showed that, in cells expressing the dominant negative FGFR1 construct, p120-catenin association with VE-cadherin was impaired (Fig. 2H). Furthermore, to confirm that the Y658 site of VE-cadherin is the real target of FGF signaling, we examined the effect of FGF inhibition on VE-cadherin mutant carrying tyrosine to phenylalanine (dephospho-mimetic) mutation of the Y658 site (Y658F). In HUVEC, while exogenously transfected wild-type (WT) VE-cadherin localized at cell-cell junctions as expected (Fig. 2I), junctional localization of WT-VE-cadherin was no longer detected in cells lacking FGF signaling (Fig. 2J, L). Y658F VE-cadherin, however, was able to rescue the FGFR1DN effect and localize at cell-cell junctions even in the absence of FGF signaling (Fig. 2K, L). Thus, we concluded that VE-cadherin phosphorylation is a downstream event of FGF signaling, and inhibition of FGF signaling increases Y658 phosphorylation, thereby disrupts VE-cadherin-based junction stability.

### FGF Signaling is Required for SHP2 Expression and SHP2-VE-cadherin Association

**Figure 3:**
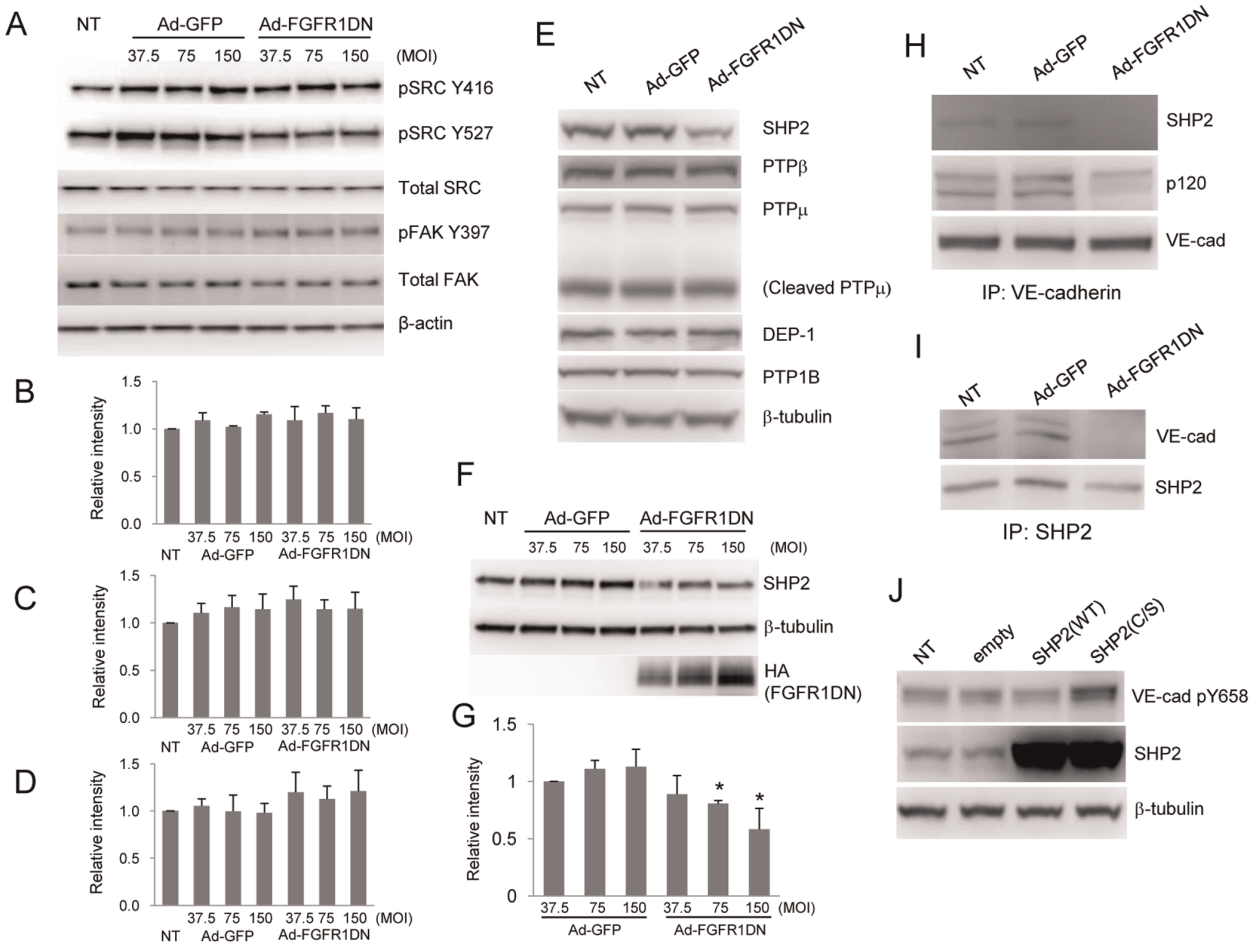
SHP2 is down-regulated and dissociated from VE-cadherin in cells lacking FGF signaling. **A.** Inhibition of FGF signaling did not activate Src or FAK. BAEC were transduced with Ad-GFP or Ad-FGFR1DN. Cells in normal growth medium were lysed and total cell lysates were subjected to SDS-PAGE followed by immunoblotting (IB) with the indicated antibodies. **B**, **C**, **D**, Quantitative analysis of pY416 Src (B), pY527 Src (C), pY397 Fak (D) shown in Fig. 3A. The value of NT, standardized with β-tubulin, was designated as 1. (n = 3). **E**, **F**. Reduced SHP2 expression in cells lacking FGF signaling. Western analysis using BAEC total cell lysates left untreated or transduced with either Ad-GFP or Ad-FGFR1DN. **G**, Quantitative analysis of SHP2 levels shown in Fig. 3F. The value of Ad-GFP at MOI 37.5, standardized with β-tubulin, was designated as 1. (n = 3 Mean ± SD, *P<0.05, by t-test compared with Ad-GFP, MOI 37.5). **H**, **I**. VE-cadherin-SHP2 interaction was disrupted by FGF signaling inhibition. BAEC were transduced with Ad-GFP or Ad-FGFR1DN. Cells were lysed and immunoprecipitated (IP) with anti-VE-cadherin (H) or anti-SHP2 (I) and subjected to SDS-PAGE followed by immunoblotting (IB) with the indicated antibodies. NT denotes no transduction. **J**. Catalytically inactive, dominant-negative SHP2 increased Y658 VE-cadherin phosphorylation. BAEC were transduced with lentivirus wild-typeSHP2 (WT) or dominant-negative-SHP2 (C/S). Cells were lysed and total cell lystes were subjected to SDS-PAGE followed by immunoblotting (IB) with the indicated antibodies.

Next we investigated the molecular mechanism of FGF-dependent regulation of VE-cadherin phosphorylation. Src has been identified as a kinase responsible for VE-cadherin Y658 phosphorylation [14]. To examine the possibility that FGF signaling modulates Y658 phosphorylation by regulating Src activity, we evaluated phosphorylation status of Src Y416 that is considered to be an indicator of Src activity. As shown in Fig. 3A, B, phosphorylation levels of Src Y416 site was not increased in cells expressing FGFR1DN in the normal culture condition. Src Y527 phosphorylation which inhibits Src activation did not change in these cells either, further suggesting that FGF inhibition does not result in Src activation that could lead to VE-cadherin phosphorylation (Fig. 3A, C). Focal adhesion kinase (FAK) is also reported to regulate VE-cadherin phosphorylation levels [38]; however, phosphorylation levels of FAK were not increased in cells lacking FGF signaling (Fig. 3A, D).

Since activity of two known kinase controlling VE-cadherin phosphorylation was not affected, we then evaluated the possibility that a PTP, but not a kinase, is responsible for modulating Y658 VE-cadherin phosphorylation. Among PTPs, DEP-1 (CD148), VE-PTP (PTPβ), PTPµ, PTP1B and SHP2 have been implicated in the interaction with the VE-cadherin-catenin complex and/or regulation of VE-cadherin function by modifying its phosphorylation status [18], [20], [21], [22], [39]. We found that suppression of FGF signaling impaired SHP2 expression while the expression levels of other PTPs were not affected (Fig. 3E, F. G). Under baseline conditions, SHP2 co-immunoprecipitated with VE-cadherin; however, expression of the FGFR1DN construct fully abolished this interaction (Fig. 3H, I).

These results suggest the possibility that SHP2 bound to VE-cadherin may regulate the phosphorylation level of VE-cadherin Y658 site. To formally evaluate this, we tested the effect of inhibition of SHP2 activity on VE-cadherin Y658 phosphorylation. Expression of the C/S mutant of SHP2 (a catalytically inactive form) in BAEC led to an increase in VE-cadherin Y658 phosphorylation compared to SHP2 overexpressing cells or control cells (Fig. 3J), while overexpression of SHP2 does not affect VE-cadherin expression levels (not shown).

### SHP2 Expression is Controlled Post Translationally by FGF Signaling

**Figure 4:**
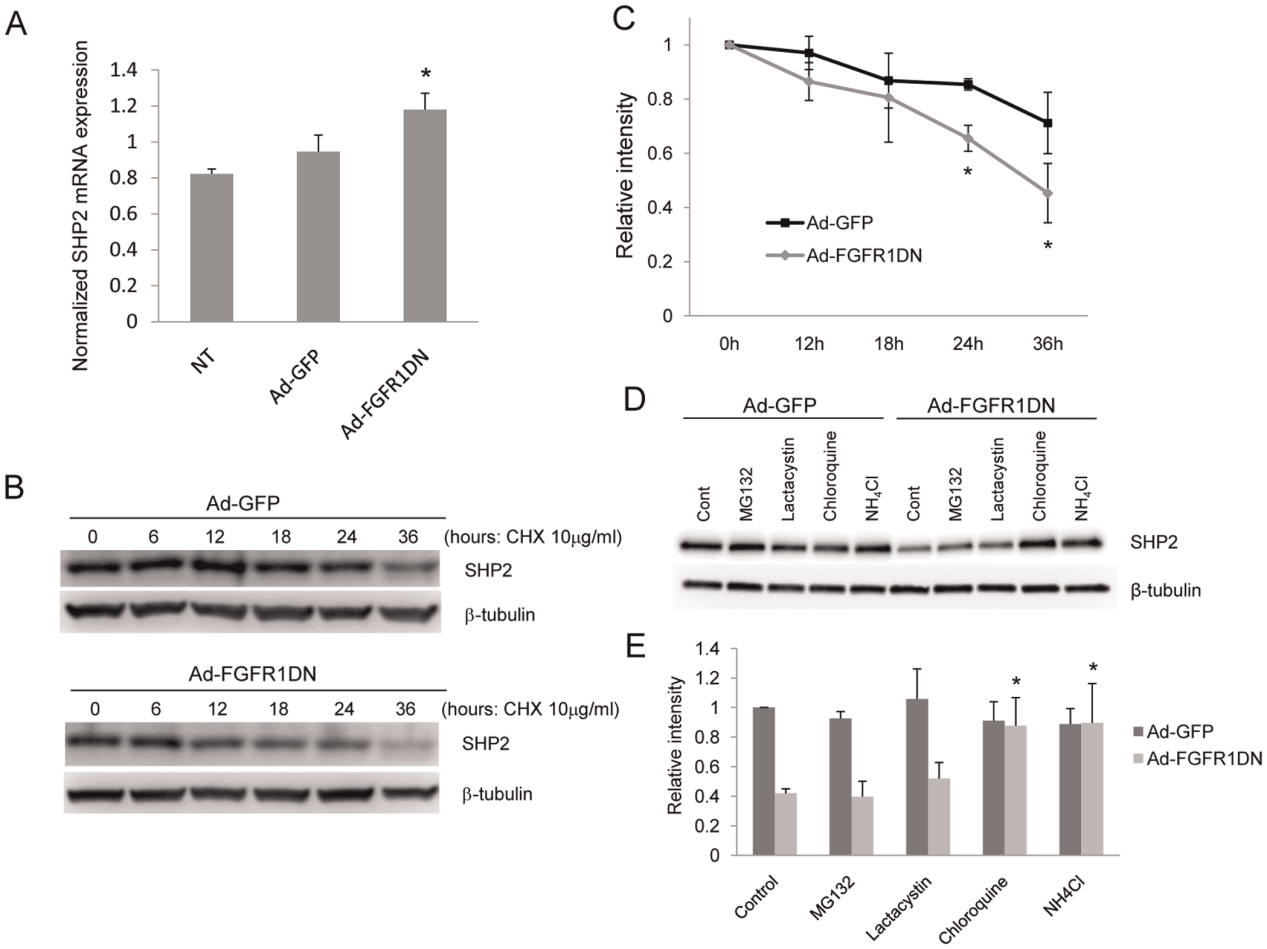
SHP2 protein stability is impaired in cells lacking FGF signaling. **A.** SHP2 mRNA levels were not decreased in endothelial cells lacking FGF signaling. Quantitative RT-PCR analysis of total RNA isolated from BAEC. Total RNA was isolated from BAEC transduced with Ad-GFP or Ad-FGFR1DN. SHP2 mRNA levels were measured with real-time PCR and normalized to GAPDH expression (Mean ± SD, *P<0.05, by t-test compared with NT). NT denotes no transduction. **B**. Western blotting of total cell lysates isolated from BAEC transduced with Ad-GFP or Ad-FGFR1DN and treated with 10 µg/ml cycloheximide for up to 36 hours. **C**. Quantitative analysis of SHP2 Western analysis described in B. The value at time point 0 was designated as 1. (n = 3 Mean ± SD, *P<0.05, by t-test compared with Ad-GFP). **D**. SHP2 is degraded via the lysosomal pathway in the absence of FGF signaling. Confluent BAEC transduced with either Ad-GFP or Ad-FGFR1DN were treated with 1 µM MG132, 20 µM lactacystin, 20 µM chloroquine or 25 mM NH_4_Cl for 24 hr. Total cell lysates were analyzed by Western blot. **E**. Quantitative analysis of SHP2 expression shown in Fig. 2D. The value of Ad-GFP control (DMSO) treatment, standardized with β-tubulin, was designated as 1. (n = 3, Mean ± SD, *P<0.05, by t-test compared with Ad-FGFR1DN control).

To determine the mechanism of SHP2 down-regulation by FGF signaling shutdown, we next tested whether FGF signaling controls SHP2 expression at the transcriptional or post translational level. Quantitative analyses of SHP2 mRNA levels using real-time quantitative PCR in BAEC transduced with Ad-FGFR1DN showed an increase in mRNA abundance while the control virus had no effect (Fig. 4A). This excludes the possibility that FGF inhibition decreases SHP2 expression through a transcriptional mechanism. At the same time, SHP2 protein half-life was shortened in cells lacking FGF signaling (Fig. 4B, C), indicating accelerated degradation of SHP2 in the absence of FGF signaling. Therefore, we concluded that FGF signaling controls SHP2 expression in endothelial cells at the post translational level. To understand the pathway of SHP2 degradation, cells transduced with either Ad-GFP or Ad-FGFR1DN were treated with proteasome or lysosome inhibitors. Decreased SHP2 expression in Ad-FGFR1DN cells was rescued by two lysosomal inhibitors including chloroquine and ammonium chloride, but not with proteasome inhibitors, suggesting that FGF inhibition accelerates lysosome-mediated SHP2 degradation (Fig. 4D, E).

### SHP2 Overexpression can Rescue Increased Permeability following Inhibition of FGF Signaling

**Figure 5:**
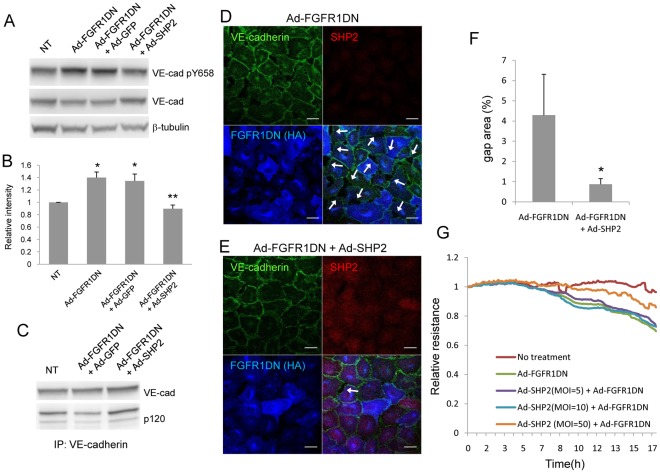
Endothelial cell phenotypes derived from FGF inhibition was rescued by SHP2 overexpression. **A.** Increased phosphorylation of VE-cadherin Y658 in cells lacking FGF signaling was restored to the basal level by SHP2 overexpression. BAEC were transduced with Ad-FGFR1DN and Ad-GFP or Ad-SHP2. Cells were lysed and total cell lysates were subjected to SDS-PAGE followed by immunoblotting (IB) with the indicated antibodies. **B**. Quantitative analysis of SHP2 shown in Fig. 5A. The value of NT, standardized with β-tubulin, was designated as 1. (n = 3 Mean ± SD, *P<0.05, by t-test compared with NT, **P<0.05, by t-test compared with Ad-FGFR1DN+Ad-GFP). **C**. SHP2 overexpression restored p120-catenin/VE-cadherin association. BAEC were transduced with Ad-FGFR1DN and Ad-GFP or Ad-SHP2. Total cell lysates were isolated and immunoprecipitated with VE-cadherin antibody. Immunoprecipitates were subjected to SDS-PAGE followed by immunoblotting (IB) with the indicated antibodies using the same membrane after stripping and reprobing. **D**, **E**, Immunostaining of quiescent and fully confluent BAEC transduced with Ad-FGFR1DN (D), or Ad-SHP2 and Ad-FGFR1DN (E). Cells were stained for VE-cadherin (green), SHP2 (red), and HA (FGFR1DN, blue). Arrows indicate gap formations between cells. **F**. Quantitative analysis of immunostaining evaluating the gap formation. Percent of gap area in each image was calculated using NIH Image J software using 6 different images. Data shown as mean ± SD *: *P*<0.05 by t-test. Scale Bars: 10 µm. **G**. SHP2 overexpression rescued the FGFR1DN effect on endothelial permeability. BAEC were transduced with Ad-SHP2 and Ad-FGFR1DN, and endothelial monolayer permeability was evaluated with the ECIS system. Transendothelial resistance was measured every 5 minutes for 17 hours after the onset of adenoviral transduction. NT denotes no transduction.

Since our observation indicates that SHP2 expression is impaired in cells transduced with Ad-FGFR1DN, we tested whether SHP2 overexpression can rescue the phenotypes of endothelial cells lacking FGF signaling. Adenoviral transduction of SHP2 into Ad-FGFR1DN-tranduced BAEC resulted in a reduction of Y658 VE-cadherin phosphorylation (Fig. 5A, B) and restoration of VE-cadherin-p120-catenin association as demonstrated by co-immunoprecipitation (Fig. 5C). In line with these observations, gap formation in the endothelial monolayer resulting from FGF signaling inhibition was suppressed by SHP2 overexpression (Fig. 5D, E, F). Finally, Ad-SHP2 transduction restored endothelial monolayer resistance that was impaired by Ad-FGFR1DN (Fig. 5G). These data indicate that SHP2 is capable of controlling VE-cadherin phosphorylation, thus restoring impaired endothelial barrier function induced by FGF signaling inhibition.

## Discussion

The results of this study demonstrate that increased permeability of the endothelial monolayer after suppression of FGF signaling is due to the loss of adherens junctions mediated by a decrease in SHP2 expression that directly leads to increased VE-cadherin phosphorylation at the Y658 site and the loss of VE-cadherin-p-120 binding. Several lines of evidence support this conclusion. Most directly, adenovirus-mediated SHP2 overexpression can rescue increased permeability, increased Y658 phosphorylation and the loss of VE-cadherin-p120 binding caused by inhibition of FGF signaling. FGF signaling controls SHP2 protein levels by inhibiting lysosome-mediated SHP2 degradation with the absence of FGF signaling input leading to a markedly shortened SHP2 half-life. Finally, the key role of Y658 site phosphorylation is demonstrated by the rescue of the Ad-FGFR1DN phenotype by Y658F VE-cadherin mutant. Taken together, these data demonstrate that FGF signaling potentiates VE-cadherin stability at endothelial junctions by regulating SHP2 ability to restrain tyrosine phosphorylation of VE-cadherin.

Many previous studies support the idea that tyrosine phosphorylation of VE-cadherin and other components of adherens junctions is associated with impaired barrier function. Permeability-increasing agents such as histamine, platelet-activating factor and VEGF all induce tyrosine phosphorylation of VE-cadherin-catenin complex [9], [10], [11], [12], [13]. Although contributions of Src, FAK and Pyk have been reported, the balance of inputs regulating VE-cadherin phosphorylation, however, remains to be fully elucidated [14], [16], [31]. The involvement of Src in VE-cadherin phosphorylation is based on the observations that it associates directly with VE-cadherin, and that VEGF cannot induce VE-cadherin phosphorylation in Src-deficient mice or wild-type mice treated with Src inhibitors [40]. Yet, the precise site of Src-induced phosphorylation in VE-cadherin is still controversial. In this study, increased VE-cadherin Y658 phosphorylation was not associated with increased Src activation, suggesting that Src was not the key player driving the observed phenotype of endothelial cells lacking FGF signaling. At the same time, there was no increase in activity of other kinase implicated in VE-cadherin phosphorylation.

These results suggest a possibility that an alternation in PTP rather than kinase activity is the principal driver of the phenotype. Unlike kinases, PTP activity is largely determined by the expression level or localization and not by a specific phosphorylation event. A number of PTPs including DEP-1, VE-PTP (PTPβ), PTPµ, PTP1B and SHP2, have been reported to directly or indirectly associate with VE-cadherin and/or adherens junction components. PTPµ is reported to localize almost exclusively at endothelial junctions where it associates directly with VE-cadherin in both human pulmonary artery and lung endothelial cells [20]. In human lung microvascular endothelial cells, PTPµ knockdown impaired endothelial barrier function, whereas overexpression of PTPµ resulted in decreased tyrosine phosphorylation of VE-cadherin and enhanced barrier function [20]. Similarly, suppression of VE-PTP or PTP1B causes endothelial barrier dysfunction [21], [39] while DEP-1 co-distributes with VE-cadherin at endothelial cell junctions and controls VE-cadherin-induced inhibition of VEGFR2 activation [18].

SHP2 has also been shown to associate with VE-cadherin via β-catenin [22]. Thrombin-induced loss of SHP2 from adherens junctions correlates with increased tyrosine phosphorylation of VE-cadherin-catenin complex, leading to adherens junction disassembly [22]. In the pulmonary endothelium, SHP2 supports basal endothelial barrier function by coordinating the tyrosine phosphorylation profile of VE-cadherin-catenin complex and p190RhoGAP and RhoA activity [41]. In line with these previous reports, we found that the loss of SHP2 from VE-cadherin in the setting of FGF signaling inhibition was correlated with increased tyrosine phosphorylation of VE-cadherin which resulted in endothelial barrier dysfunction.

**Figure 6:**
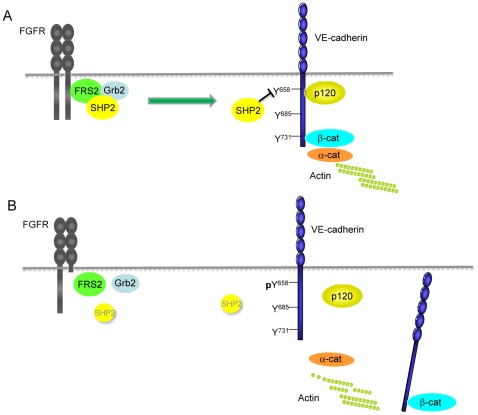
FGF signaling is required for VE-cadherin-SHP2 interaction which stabilizes adherens junctions. **A.** Under normal circumstances, SHP2 is associated with VE-cadherin and dephosphorylates Y658 site of VE-cadherin, which results in p120-catenin coupling, enhancing VE-cadherin retention at adherens junctions. FGF signaling is required for VE-cadherin-SHP2 interaction. **B**. In cells expressing the FGFR1DN construct, SHP2 is downregulated and is not able to associate with VE-cadherin due to the lack of FGF signaling, which increases phosphorylation level of VE-cadherin Y658, leading to decoupling of p120-catenin; therefore, VE-cadherin stability at cell-cell junctions is impaired.

How SHP2 is recruited to VE-cadherin-catenin complex and which tyrosine phosphorylation of VE-cadherin is regulated by SHP2 is of special interest. One study shows that SHP2 is recruited to VE-cadherin when VEGF receptor tyrosine kinase phosphorylates VE-cadherin. The VE-cadherin-bound SHP2 induces release of Csk from VE-cadherin potentially by dephosphorylating its VE-cadherin binding site (Y685). Consequently, c-Src Y527 phosphorylation level decreases, and c-Src Y416 phosphorylation level increases which in turn results in c-Src activation [42]. In the current study we observed that SHP2 was associated with VE-cadherin in resting cells. In cells lacking FGF signaling, SHP2 dissociated from VE-cadherin and the phosphorylation level of VE-cadherin Y658 increased while phosphorylation levels of Y685 or Y731 were unchanged. Moreover, overexpression of the C/S mutant of SHP2, a catalytically inactive form of SHP2, in endothelial cells enhanced Y658 phosphorylation of VE-cadherin, indicating SHP2 is necessary for dephosphorylating the Y658 site. Taken together, we propose that SHP2 associates with VE-cadherin in resting cells, which maintains the Y658 site dephosphorylated, leading to the enhancement of endothelial junction stability, and basal FGF signaling is required for this VE-cadherin-SHP2 interaction (Fig. 6).

While we show that FGF signaling is required for VE-cadherin-SHP2 interaction, certain aspects of this regulation remain uncertain. We found that downregulation of SHP2 expression in the absence of FGF signaling is due to shortened protein half-life and that inhibition of the lysosomal pathway, but not the proteosomal pathway, restored the SHP2 expression levels of cells lacking FGF signaling. Although we also examined the contribution of autophagy in this process, we were not able to obtain the evidence suggesting that FGF signaling controls autophagy in endothelial cells. Therefore, we concluded that SHP2 is degraded via lysosomal pathway in the absence of FGF signaling; however, how FGF controls this process is unclear. Furthermore, the precise mechanism responsible for FGF-dependent association with SHP2 with VE-cadherin is uncertain. One possibility is that the latter can occur via the VEGF signaling pathway since VEGF can recruit SHP2 to VE-cadherin-catenin complex [42], [43]. We have recently demonstrated that VEGF receptor 2 expression is critically dependent on continuous FGF stimulation [36]. Thus, in the absence of FGF input VEGF signaling is down-regulated, which in turn impairs SHP2 recruitment to VE-cadherin.

Finally, it is unclear which FGF is responsible for continuous stimulation of the endothelium necessary for the maintenance of SHP2 expression. Endothelial cells express all four FGF tyrosine kinase receptors and numerous FGF ligands are present in the serum in vitro and in blood and interstitial fluid in vivo. Given significant redundancy in the FGF signaling pathways where multiple ligands can activate multiple receptors, it is virtually impossible to identify a specific FGF that can be responsible for this effect. In fact it is likely that multiple redundant ligands are capable of performing this vital function.

In summary, we report a novel function of FGF signaling in the vasculature that involves FGF-dependent maintenance of endothelial SHP2 levels and plays a key role in the regulation of endothelial barrier function.
